# Beyond the plate: Exploring students’ experiences with a food education intervention in an after-school program setting

**DOI:** 10.1016/j.puhip.2026.100815

**Published:** 2026-05-23

**Authors:** Cecilie Beinert, Anita Nistås, Eli Anne Myrvoll, Frøydis Nordgård Vik, Margrethe Røed

**Affiliations:** Department of nutrition and Public Health, Faculty of health and Sports Sciences, University of Agder, Norway

**Keywords:** Food education, After-school programs, Students, Experiences

## Abstract

**Objectives:**

Establishing healthy dietary patterns in childhood lowers the risk of developing non-communicable diseases. After-school programs are important settings in public health work, as they reach a majority of young students and shape everyday food and meal practices.

The aim of this study was to explore students’ experiences of being “food agents”, a central role in an intervention aiming to increase food literacy competencies. The after-school program employees' experiences with students as food agents during the intervention were included in the analysis, to broaden the perspectives.

**Study design:**

Qualitative focus groups and individual interviews.

**Methods:**

Qualitative focus groups with 19 students from 2nd (7-8 years old) to 4th grade (10-11 years old) and individual interviews with six after-school program employees in Norway. The anonymous transcripts were uploaded to Nvivo 15 and analysed inductively using Braun and Clarkes reflexive thematic analysis approach.

**Results:**

The students enjoyed being part of the intervention and acting as food agents. They reported joy of preparing and serving food, learning new cooking skills and trying new foods. Gaining responsibility and receiving positive feedback from peers on their work is something which provided them with a sense of growth and mastering. This feedback was supported by the staff who observed positive engagement among the students.

**Conclusions:**

Our results suggest that including students in preparing and serving meals in after-school programs can provide students with valuable experiences related to both food literacy competencies and personal growth.

What this study adds.

• This study supports a continued and consistent focus on healthy food and meal practices in after school programs as part of the cross-sectoral approach to public health work.
• This study demonstrates improved food literacy among students who are included in the practical components of food preparation in after school programs.

Implications for policy and practice.

• Our study results can inform after school program leaders and employees to include students in meal preparation to support students' food literacy competences.
• Our results indicate that after school program guidelines should emphasize not only what food is served, but also the importance of involving students in practical food-related activities.

## 1 Introduction

A WHO-Lancet commission acknowledges childhood as “a special time for vulnerability, but also of opportunity” [1]. Establishing healthy dietary patterns in childhood will have important health benefits as it reduces the risk of developing non-communicable diseases [2,3]. Bundy et al. [4] argue that a focus on the first 1000 days is important, but not sufficient, and calls for interventions in what they call the middle childhood growth and consolidation phase (5-9 years), adolescent growth spurt (10-14 years), and adolescent growth and consolidation phase (10-19 years).

Food education interventions in school and after-school programs (ASPs) that include practical cooking lessons have been regarded especially effective in increasing confidence regarding food and cooking, but also for creating behavioural change [5,6]. A literature review of primary school experiential nutrition programs found multi component interventions to be most effective on cognitive and behavioural outcomes [7]. In the same study, cooking-based activities were shown to be most influential in increasing self-efficacy, food knowledge, and food preferences. Another systematic review on different types of interventions found that including practical cooking lessons to be positive in improving cooking knowledge and cooking behaviour amongst school aged children, although more rigorous interventions and measurement instruments are needed [8]. All the above-mentioned interventions are central in building food literacy competence in students.

There are many definitions of food literacy, but they share the common trait of encompassing both practical and theoretical knowledge and skills [9]. Benn [10] has broadened the term and included the verbs knowing, doing, sensing, wanting, and caring. Food literacy emphasizes knowledge and skills, but also sensory experiences, food courage, and the willingness to take responsibility and care for others through food [10]. Food literacy has been recognized as an important health determinant at the individual level, as it empowers people to make healthy and sustainable dietary choices [11]. Development of functional competencies like nutrition knowledge, cooking skills and food safety knowledge often starts at the age of 6 to11 years [12], making schools and ASPs an ideal setting for working with such food related competencies. Students enjoy cooking based activities [13], which is a good starting point when working with this age group. If an activity is enjoyable, it is more probable that individuals will have intrinsic motivation, which, according to Deci and Ryan [14], leads to increased motivation.

In 2024, 95% of first graders, 86% of third graders and 37% of fourth graders attend ASPs in Norway [15]. According to the ASP Framework Plan, which is a regulation of the Education Act, ASPs shall facilitate daily mealtimes [16]. These meals should follow national dietary guidelines and emphasize sustainable eating habits and consumption patterns. It also states that preparing meals together with the students can provide them with experience of how to prepare basic and healthy meals and create a pleasant environment around mealtimes. Still, ASP leaders have reported difficulties following the guidelines for food and meals in ASPs mainly due to limited human and economic resources [17,18].

Previous research on food and meal practices in Norwegian ASPs is sparce. A national evaluation of ASPs in Norway in 2018 revealed that food and meals is a demanding area for ASPs, and there are many employees and managers who find food handling procedures problematic [19]. Furthermore, Caspersen and colleagues [18] identified that time, budget and interests among ASP employees strongly influence what is served in ASPs. Also, a positive correlation between participating in competence development measures and ASP employees' own assessment of whether they have worked differently after the introduction of the new framework plan in 2021 has been identified [20]. This supports the assumption that increased competence is important for creating change, also within food and meal practices. Finally, a gap between what is expected in the Framework plan, and the competence some ASP employees possess, has been reported by ASP leaders [20].

We have previously investigated how ASP staff and leaders experience this educational intervention provided by Geitmyra Culinary Centre for Children (Geitmyra for short) [21]. However, students’ experiences remain unexplored. Within this educational intervention, the older students in ASPs are trained as “food agents”, having responsibility for preparing, presenting and serving various dishes to the others students.

The aim of this study was to explore students' experiences of being “food agents”, a central role in the intervention. Our research question was: how do students experience the role of being food agents in an educational intervention developed and implemented by Geitmyra, aiming at improving food literacy competencies? We also included the ASP employees' experiences with students as food agents during the intervention, as a supplement to those of the food agents to broaden the perspectives and understanding of the food agent's role.

## 2 Methods

### 2.1 About the intervention

Geitmyra is a Norwegian non-profit foundation [22], offering a specific educational intervention for staff and students in ASPs in a county in southern Norway. All ASPs are required to adhere to standards set by Geitmyra and include key components and dates laid out in Table 1.The primary aim of this intervention was to improve food literacy amongst the ASP staff and students, and thereby ensuring the ASPs provide a nutritious menu that adheres to national dietary guidelines.

**Table 1:** Key components of Geitmyra Culinary Centre for Children's intervention in after-school programs (ASPs) year 2024.

Time period	Key components	Participants
June	Digital information meeting to motivate for participation	ASP leaders
Aug/Sep	Startup meetings (at ASP and/or digital	School principals
		ASP staff (incl. leaders)
Aug/Sep	Students apply for the role of food agents	ASP staff (incl. leaders)
		Parents
		Students (mostly, 3rd graders, 8-9 years)
Sep/Oct	Culinary training at Geitmyra (3hrs)	ASP staff (incl. leaders)
Sep/Oct	Culinary training at Geitmyra (3hrs)	ASP Staff
		Food agents
Sep/Oct/Nov	Visits at ASP for further guidance, cooking classes, and meals (approx. 6 visits)	ASP staff
		Students
		Food agents
Nov/Dec	Food festival at ASP hosted by ASP and Geitmyra	ASP staff and food agents
		School staff and students
		Local community

Geitmyra emphasizes the importance of adjusting goals and components to each ASP's local contextual factors, aiming to overcome common barriers for implementation, such as limited time available, constrained financial resources, or lack of proper kitchen facilities and/or equipment. One of the key components of the intervention is that some of the students, typically 3rd graders (8-9 years old), are trained in the role of “food agents”. Some ASPs, particularly those with a reduced number of students, also include 2nd (7-8 years old) and/or 4th graders (10-11 years old). The purpose of the food agents is to be involved in both cooking and facilitating the meals in their ASP. Their tasks vary between ASPs due to local contextual factors at the ASP, but typically they help prepare and serve hot meals at least once a week and help with various kitchen-related tasks during the week, such as preparing fruits and vegetables, making crispbread, setting the table, or tidying up. At some ASPs, food agents also help in the school garden, like growing vegetables. Most importantly, they are to serve as role models, promoting healthy eating habits, food courage, and the joy of food to their peers. The students apply for the role as food agents, and the teacher selects a group based on their motivation for the role.

Both ASP staff and food agents attend nutrition and culinary training courses held at Geitmyra's facilities. Employees are informed about the purpose of the intervention and the importance of adherence to the national dietary guidelines [23] and the ASP Framework Plan [16]. This is done through practical examples aiming to raise awareness of the courage to taste new food, the importance of healthy eating habits, and children's dietary needs. Geitmyra promotes discussions on how to contribute to a pleasant mealtime environment and foster enjoyment of food and thereby raises awareness of the meal as an important social activity. The final component of the intervention introduces recipes developed by Geitmyra and includes practical cooking activities, culminating in a shared meal. The food agents get a similar course adjusted to their age group. This course clarifies expectations for the role of food agents, introduces the five basic tastes, addresses food waste, and outlines kitchen hygiene and safety rules. In the final part, the food agents are taught basic cooking skills and participate in practical cooking activities, using the same recipes as the ASP staff. The course culminates in a shared meal and a conversation about taste and favourites from the menu.

As a standard, Geitmyra visits the ASPs six times during one semester for further practical cooking classes for staff and food agents, guidance according to local contextual factors and menu adjustments. In addition, the ASP can contact Geitmyra for further guidance by phone or e-mail. At the end of the semester, Geitmyra and the ASP invite all students, parents, and staff to join a food festival. Some ASPs invite the whole local community or county. At the festival, adults and students from the ASP and school engage in cooking activities and get to taste some of the food served at the ASP. The food agents act as co-hosts, planning and conducting the activities at the festival along with the staff from Geitmyra and the ASP.

### 2.2 Data collection

Qualitative semi-structured interviews were conducted with both ASP staff and students (food agents) at three different schools in two municipalities in southern Norway, gaining in-depth insights and understanding experiences associated with the role of food agents [24] (see Table 2). To enhance the overall understanding of students’ experiences as food agents, interviews with ASP employees who worked alongside the food agents were incorporated into the analysis to capture their perspectives on how the intervention influenced the students.

**Table 2:** Description of participating ASPs, method for data collection and participants.

ASP	Type of interview	Participants
ASP 1 Rural area Approx. 35 students Attended autumn 2024	Semi-structured focus group	8 students (2nd – 4th grade, 7-10 years old, 5 boys + 3 girls)
	Semi-structured individual interviews	2 employees
ASP 2 Small town Approx. 100 students Attended spring 2024	Semi-structured focus group	4 students (3rd grade, 8-9 years old, girls)
	Semi-structured Individual interviews	2 employees
ASP 3 Small city Approx. 120 students Attended spring 2024	Semi-structured focus group	7 students (3rd grade, 8-9 years old, girls)
	Semi-structured Individual interviews	2 employees
**In total**		**Students n = 19**
		**Employees n = 6**

Students participated in semi-structured focus groups while employees participated in individual interviews. Both were recruited through written and oral information given at the ASP by the interviewer (X4) and the ASP leaders. Written, informed consent was obtained from each employee and parent/care giver (for students) prior to the interviews. Students received age-adjusted written information and provided verbal assent to participate.

The students were interviewed in designated small meeting rooms at their ASP during mealtimes. The interviewer participated in the meal preparation to get to know the food agents. ASP staff were nearby during the interview, helping the students feel more comfortable. Individual interviews with ASP staff were conducted in person at their ASPs. Both the individual interviews and focus groups lasted 30-40 min. Two semi-structured interview guides were developed, exploring experiences with the intervention and the use of food agents. The students’ interview guide was modified to fit their age.

The interviews were recorded using Nettskjema dichtaphone application (University of Oslo, 2025, https://nettskjema.no/) and then transcribed intelligent verbatim [24]. Any communication between students and ASP staff, who entered the room to serve the meals during the focus group, was omitted. Brackets were used to include relevant body language or contextual information in the transcripts. All students and employees were assigned pseudonyms to ensure confidentiality.

### 2.3 Data analysis

A phenomenological approach was adopted in this study. Phenomenology can be described as the exploration of a given phenomenon through the shared, lived experiences of several individuals [25]. The aim was to distil these experiences, in this case ASP students' and employees' experiences with a specific role of food agents, into a description of the universal essence of the intervention under exploration.

The anonymous transcripts were uploaded to Nvivo 15 (Lumivero, Denver, CO) for data management. Data was analysed inductively by the first author using Braun and Clarkes [26] reflexive thematic analysis approach, which was regarded suitable for exploring students' experiences. This analysis approach is flexible, and well suited to develop and develop rich, qualitative descriptions which can provide new insight into a phenomenon [26].

There are six phases of reflexive thematic analysis. The researchers first read and reread each transcript to get an initial overview, while making initial notes. The student interviews were read first, then the employees, before all interviews were analysed. Sections relevant to the research question were then coded by both the first and second author (who conducted the interviews) separately. After the initial coding was conducted, these were discussed between the two authors, which provided us with a deeper engagement and more nuanced analysis. Possible initial themes were discussed based on the findings. Although qualitative analysis does not aim to capture an objective “truth”, working towards a “fit” between respondents' views and the researcher's representation of them will increase the trustworthiness of the findings [27]. After revisiting the codes, some codes were deleted because they were not regarded significant, some content was recoded into different codes, some were collated into themes, and some were created as a theme themselves, consisting of several child codes. As an example, the code “learning safe handling of kitchen tools” was merged into the code “learning new cooking skills”, which became part of the theme “learning new skills and values”. “It was fun!” was a code which became a theme itself. Throughout the analysis, there has been a reflexive process of moving back and forth between each phase in generating codes and themes [28]. As Beinert, Røed and Vik had explored employees' experiences with this intervention in a previous study [21], it was important to be mindful of potential assumptions through the analysis process.

## 3 Results

The following four themes were identified during the analysis; It was fun!, growth through experience, ripple effects, and learning new skills and values.

### 3.1 It was fun!

The most prominent feedback from the food agents was that the experience of participating in the programme as a “food agent” was fun, that they enjoyed handling food and making meals, and that they enjoyed doing something new. The enjoyment of cooking itself was the main feedback, but the fun serving food to the other students was highlighted as a positive experience. One food agent described the experience of being a food agent positively when asked about their role: “*It's been really fun, because we get to cook and it's fun when we learn something new*” (“Nina” ASP 2, 3rd grade). The word “fun” was significant throughout the interviews with the food agents, when being explicitly asked how it has been for them to participate. This experience was supported by the ASP employees. As one employee stated when asked how the students behave in the kitchen preparing the food; “*Very, very good. Very proud of the job they have and what they are doing. It just becomes this immense joy of being in the kitchen together. And yes, they argue over who gets to be there*” (“Vera”, ASP 2). The enjoyment of cooking, learning new things and serving food are closely linked to the next theme.

### 3.2 Growth through experience

This theme is about the feeling the food agents got when receiving positive feedback from peers, and both getting and taking responsibility throughout the intervention. In combination, these experiences may provide food agents with the feeling of growth and development as human beings. A few of them described how they took control and corrected other students if they were especially noisy during the mealtime, or if they spoke poorly about the meal they were served. By being in charge of preparing the meal and serving the younger students at the ASP, the food agents gained valuable experiences and responsibility related to the mealtime. Instead of being passive receivers, food agents take an active role in helping to create a pleasant mealtime. As illustrated in an interview at ASP 1 between the interviewer and a 4th grade food agent:

“Interviewer: *How has it been serving the others food?*

“Kristen”: *A bit cozy actually*

Interviewer*: A bit cozy, tell me why you think that is?*

“Kristen”: *I feel a bit more like a man, or yes, grown up when I get to serve the food instead of just sitting in my seat waiting”.*

This student explained how he felt more like a grown up when being a food agent. This experience of mastering and being helpful is something one employee,“Anne” at ASP 1, highlighted when asked if she believed there has been any ripple effects outside just preparing the food: “*Yes, I believe they have gotten – like they have grown a bit, because they have been shown that responsibility. And then us adults* ^have^*, at least I believe we have, been very good at talking positive about them (the food agents) to the rest of the school …”.*

The food agents received positive feedback from peers, which provided them with the feeling of mastering and giving joy to others, as illustrated here when a 3rd grade food agent at ASP 2 is asked how it is like serving the other students food.

Mali: “*If they don’t know who made the food, and you eat at the same table, it’s quite fun when they say ”yummy, that’s tasty*””.

### 3.3 Learning new skills and values

As a practical cooking course, the food agents spend most of their time preparing different dishes that are served to the other students. When asked what they have learned during the intervention, many students highlight new cooking skills, like how to hold and cut with a knife in a safe way. They refer to it as the “claw” and the “tunnel”, as exemplified by food agent “Nina”, at ASP 2:

*“*Interviewer*: Do you have any examples of things you have learned? Yes, please go, Nina.*

Nina*: Knife grip!*

Interviewer*: Can you explain it to me, and use words to explain what that is?*

Nina*: When you cut like lengthwise, then we hold like, this tunnel [shows with fingers]. And if we shall cut like up and down, then we hold it like a claw [shows with fingers]”*.

This example illustrates how the food agents have learned quite specific and easy to remember cutting techniques. The students mention different dishes they have learned how to make themselves from scratch, like homemade fish fingers, broccoli soup, baked vegetables, crispbread, butter, mayonnaise, and bean stew. Some students tasted these for the first time. They also talk about different manners they learned, like not screaming, washing your hands before eating, and not starting to eat before been told so. Not saying “ugh” to the food is something that many students remember, and which has been an important focus by Geitmyra:

Interviewer: *“… And they (Geitmyra) are very committed to not saying “ugh” and things like that. How is that going?*

ASP employee “Marthe", ASP 3: *We are very good at that. I see when we were in third grade, then there was this comment, and then [the food agents] immediately said; it’s not allowed to say that word. It's forbidden! Then the others started to think a little. So, they are very, at least the food agents, aware of that”.*

Still, not all that is learned was followed by the students. Employee “Amalie”, ASP 1, describes how the students behaved better when Geitmyra was there, compared to when the intervention period was finished, and they were working on their own. When asked how it has been for the employees to get food agents, “Amalie” answers: *“It's fun to see when they are so eager. But it's very different when you are here and not.*

Interviewer*: Yes, what do you think is the difference?*

“Amalie”*: When I have had them alone, there are a lot of them who - I'm trying to get them started in a way, but then there are just howling and they start to joke around …”*

### 3.4 Ripple effects

By being a food agent, the students must taste the food they serve. This way, the students build courage to taste new food, and it might widen the repertoire of the foods they like. The students mentioned several food items or dishes they had never tasted before, like cauliflower and beans, and discovered food they did not know they liked, like homemade fish fingers and stew with beans. Employee A “Marianne” at ASP 2 observed the food agents being role models in tasting new foods, when asked what has changed after the intervention:

“Marianne”*: “The kids are very positive towards the food. They eat food I never thought I could serve in a way. They are very brave tasting, and very curious.*

Interviewer*: Does this apply to the food agents, or all (students)?*

“Marianne”*: All, because the food agents have been properly trained in tasting new things and gotten used to it in a way. It was a lot of scepticism in the beginning, but now they are used to getting soups and stews with all kinds of different things in them”.*

Besides tasting new dishes and foods, some food agents talk about how they make the food they prepare at the ASP at home or that the parents prepare the different dishes, having direct impact in the homes:

“Mali”, Food agent, ASP 2: *“At home, mom has been so curious of what we have made at the ASP, at the food agent course, so she has gone online and found it there, the food agent things, recipes and stuff. So, then she has made the things we made”*. In the interview with an employee at the same ASP, they state that the ASP leader has sent an email to the parents with the recipes and that the parents have asked for them.

The food agents also talk about how they have taught cooking skills to their parents, like safe and effective ways of using a knife or how to prepare dishes from scratch, like homemade fish fingers.

## 4 Discussion

This paper explores students' experiences with being food agents, as part of an educational intervention in ASPs in Norway. The results indicate that the students enjoy being part of the intervention. “Fun” was a word most frequently used to describe how it had been participating in the intervention - it was most used to describe practical element of cooking. Cooking based activities have been regarded as a valuable food literacy skill to target [5,6], as practical cooking skills have been considered important for increasing children's self-efficacy, food knowledge and preferences [7]. This aligns with our findings, where several food agents reported making the food at home themselves after the intervention. This may have a direct impact on the quality of the food made at home and thus the diet of the family, having potential positive ripple effects. The age group included in our study has been regarded as an important target group in the development of skills related to cooking [12]. As noted by Deci and Ryan [14], if an activity is enjoyable, it is more probable that individuals will have intrinsic motivation, which in turn may lead to increased motivation. This may explain why the students reported trying out the recipes at home. Additionally, when students are aware that they need to teach or present something to others, their engagement in the learning process is heightened [29]. Our results show that the employees noticed this engagement among the students during the intervention, which may strengthen the possibility that the students adopt what they have learned to their everyday life.

Involving the students in the meal time, from cooking to serving, is something the students in this study appreciate, and is in line with the ASP Framework plan [16]. Our results indicate that the students gain valuable experience by being given responsibility and tasks to perform. In our previous publication, employees reported a positive experience regarding inclusion of students, as it lessens their workload and thus provides them with more time with the student group as a whole [21].

The willingness to take responsibility and care for others through food have been regarded as important components of food literacy, as they broaden ones perspective, encompassing life outside the individual's existence [10]. They foster motivation, as a meaningful asset to one's environment [29]. Furthermore, working as food agents may provide the students with a sense of autonomy, competence and relatedness, important human needs for motivation and growth, according to Deci and Ryan [14]. During the intervention, the students reported tasting and enjoying several food items and dishes new to them. Being introduced to tasty and healthy dishes can be important from a public health perspective, as diet during childhood and adolescence set the foundation for health later in life [[1], [2], [3]]. Including the students in preparing food and facilitating them in talking highly about the food and their experience to their peers, can have a positive social impact on attitudes towards the food. Cooking based activities have been regarded as influential in impacting behavioural outcomes related to food literacy, like food knowledge, self-efficacy, preferences, and confidence [[5], [6], [7], [8]]. Thus, finding new ways of including students in such activities in schools and ASP, as this current intervention exemplifies, can provide students with valuable experiences increasing their food literacy, and thereby their health. Even though the students describe learning new skills or being introduced to new, healthy and tasty food items, it is not guaranteed that they will continue this practice in their everyday life. As the food agents typically take full responsibility for preparing and serving a full, hot meal once a week, and not daily, the long-term effect might be diminished. Still, the students act as food agents for one full year, performing daily activities like cutting fruit and vegetables for a platter, setting the tables, and being a positive role model during mealtimes, supporting continuity.

In summary, students report having fun doing practical work like cooking and serving food, learning how to use a knife while handling food, getting positive feedback from peers, and enjoying new, healthy dishes. Considered collectively, these elements can indicate increased food literacy, regarding competencies such as practical skills and food liking, and furthermore a heightened self-efficacy amongst the students as they experience a sense of mastering different tasks.

The strength of this study is the inclusion of students' voices. Both the UN Convention on the Rights of the Child and the Norwegian ASP framework plan emphasize the students' right to participate and have an opinion on matters that affect their daily life [16,30]. Including students' voices in research can provide unique insight into their experiences with interventions they are a central part of, compared to only exploring adult experiences. Furthermore, the employees' reflections on how they experienced the students in the process of being food agents contribute to deeper insight into the phenomenon explored. The students' interviews took place in a familiar setting at their ASP and were conducted during meals in groups. Furthermore, the interviewer was allowed to prepare these meals alongside the students. These measures were taken to make the students feel more at ease and comfortable during the interviews. The analysis of the interviews was partly conducted by two of the authors following Braun and Clarke's [31] step by step approach, strengthening the trustworthiness of the findings.

Still, there are several limitations to consider. We included only three ASPs, and therefore a limited number of participants. The ASPs also varied in size and were located in different municipalities. The generalizability of the findings is therefore unclear. Also, the focus groups with the students were of various sizes because of practical reasons (n = 4 to 8). The students were quite often unfocused during the focus groups, and the interviewer therefore had to ask the questions more direct and sometimes leading, which may have impacted on their answers compared to a more open approach. Finally, it is important to note that the food agents included here were a motivated group of students. Therefore, exploring how to include a broader group of students in such interventions would be valuable. More research is of course needed to draw any strong conclusions.

### 4.1 Implications for research, practice, and policy

Developing students’ food literacy and providing students with nutritious meals daily can positively influence both short- and long-term health, making this highly relevant from a public health perspective. Involving older students in preparing and serving the meals to the younger students offers meaningful life experiences, which can increase their food literacy. Our findings indicate that students at this age enjoy participating in food-related activities, representing an important resource to build upon. However, meaningful change in ASP food practices depends on ASP leaders and employees. Previous studies have shown that staff participation in competence-building initiatives leads to positive changes in how food and meals are managed [20,21]Such approaches might also be applicable to other interventions aligned with national food and meal guidelines. Given that time, budget and interests strongly influence what is served in ASPs [18], competence-building efforts be adapted to local conditions and support motivation for change among ASP staff and leaders.

## Author contributions

This study is part of an evaluation of the educational intervention conducted by Geitmyra, managed by Røed, Beinert and Vik (project leader). Master student Nistås was responsible for recruitment of participants, conducting the interviews, and transcribing the data under supervision of Røed and Myrvoll. Beinert and Nistås wrote the initial draft of the introduction and methods section. **B**einert was responsible for analysing the data and discussing the initial codes identified with Nistås, and Beinert drafted the results and discussion section. Geitmyra delivered the intervention, but did not take part in planning or writing this paper. All authors approved the final draft.

## Funding

This study is funded by Sparebankstiftelsen, Sørlandets Kompetansefond and the University of Agder. The financial contributors were not involved in designing the study, data collection, analysis, interpretation of data, or in writing the manuscript. Open access funding provided by the University of Agder.

## Ethics statement

The study was approved by Norwegian Agency for Shared Services in Education and Research (ref. 231769) and by the faculty ethics committee and has been conducted in line with the Helsinki Declaration of 1985, revised in 2008.

## Declaration of generative AI use

Scopus AI was used in the initial search for relevant literature on after-school program interventions, and we have used the language model GPT-4 via GPT UiO to explore suitable titles, to improve sentence structure and get suggestions for synonyms to avoid repetition. After using GPT, the authors reviewed and edited the content as needed and take full responsibility for the content of the published article.

## Declaration of competing interest

The authors declare that they have no known competing financial interests or personal relationships that could have appeared to influence the work reported in this paper.

We thank all participants for sharing their experiences.
